# Pineal Neurosteroids: Biosynthesis and Physiological Functions

**DOI:** 10.3389/fendo.2020.00549

**Published:** 2020-08-11

**Authors:** Shogo Haraguchi, Kazuyoshi Tsutsui

**Affiliations:** ^1^Department of Biochemistry, Showa University School of Medicine, Tokyo, Japan; ^2^Graduate School of Integrated Sciences for Life, Hiroshima University, Hiroshima, Japan

**Keywords:** allopregnanolone, 7α-hydroxypregnenolone, neurosteroid, pineal gland, cerebellum, light

## Abstract

Similar to the adrenal glands, gonads, and placenta, vertebrate brains also produce various steroids, which are known as “neurosteroids.” Neurosteroids are mainly synthesized in the hippocampus, hypothalamus, and cerebellum; however, it has recently been discovered that in birds, the pineal gland, a photosensitive region in the brain, produces more neurosteroids than other brain regions. A series of experiments using molecular and biochemical techniques have found that the pineal gland produces various neurosteroids, including sex steroids, *de novo* from cholesterol. For instance, allopregnanolone and 7α-hydroxypregnenolone are actively produced in the pineal gland, unlike in other brain regions. Pineal 7α-hydroxypregnenolone, an up-regulator of locomotion, enhances locomotor activity in response to light stimuli in birds. Additionally, pineal allopregnanolone acts on Purkinje cells in the cerebellum and prevents neuronal apoptosis within the developing cerebellum in juvenile birds. Furthermore, exposure to light during nighttime hours can cause loss of diurnal variations of pineal allopregnanolone synthesis during early posthatch life, eventually leading to cerebellar Purkinje cell death in juvenile birds. In light of these new findings, this review summarizes the biosynthesis and physiological functions of pineal neurosteroids. Given that the circadian rhythms of individuals in modern societies are constantly interrupted by artificial light exposure, these findings in birds, which are excellent model diurnal animals, may have direct implications for addressing problems regarding the mental health and brain development of humans.

## Introduction

Similar to the gonads and placenta, vertebrate brains actively also produce various steroid hormones. These steroid hormones produced in the brain are named “neurosteroids.” The production of neurosteroids was demonstrated firstly in mammals, and then in other vertebrates (1–5). Thus, neurosteroid production appears to be a universal feature of the brain in vertebrates.

It is known that neurosteroids are produced in glial cells and neurons of the central and peripheral nervous systems (1, 5). However, we have demonstrated that the pineal gland produces neurosteroids from cholesterol in birds during early posthatch period (6–8). Notably, allopregnanolone (also known as 3α,5α-tetrahydroprogesterone; 3α,5α-THP) and 7α-hydroxypregnenolone are the two major neurosteroids produced in the pineal gland (6, 7). Of these two, pineal allopregnanolone prevents the death of developing Purkinje cells (7, 8), and pineal 7α-hydroxypregnenolone functions as an up-regulator of locomotion, regulating locomotor activity in response to light stimuli in birds (6).

## Biosynthesis of Pineal Neurosteroids

**Figure 1:**
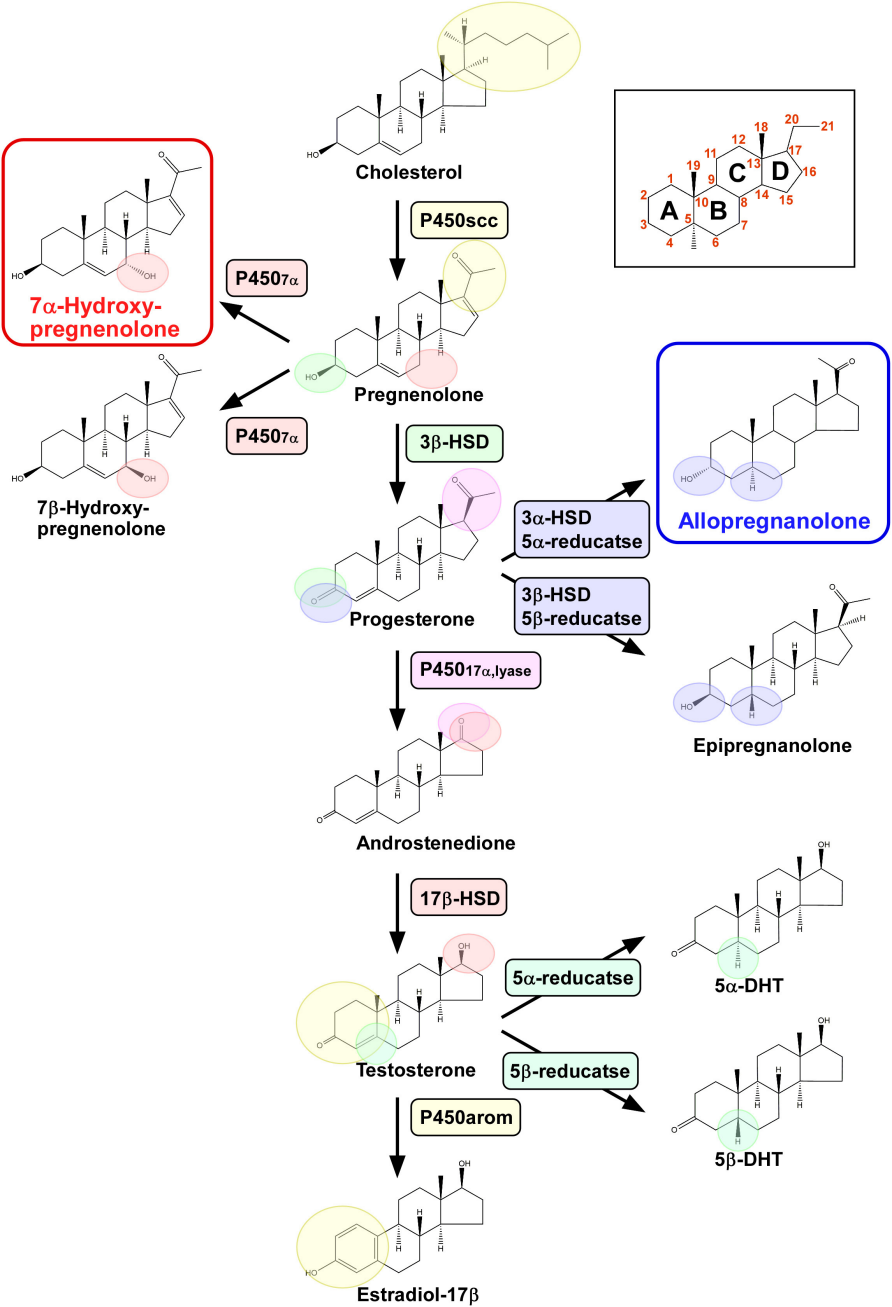
Biosynthetic pathways of pineal neurosteroids. Allopregnanolone and 7α-hydroxypregnenolone are the major neurosteroids produced in the pineal gland of birds. P450scc, cytochrome P450 side-chain cleavage enzyme; P4507α, cytochrome P450 7α-hydroxylase; 3β-HSD, 3β-hydroxysteroid dehydrogenase/Δ^5^-Δ^4^-isomerase; 3α-HSD, 3α-hydroxysteroid dehydrogenase/Δ^5^-Δ^4^-isomerase; 5α-reductase; 5β-reductase; P45017α,lyase, cytochrome P450 17α-hydroxylase/c17,20-lyase; 17β-HSD, 17β-hydroxysteroid dehydrogenase; and P450arom, cytochrome P450 aromatase.

The pineal glands of vertebrates respond to light stimuli and fulfill important functions in the organization of circadian rhythms. The secretion of melatonin, a major hormone produced by the pineal gland, shows a clear daily rhythm with its peak concentration occurring at night (7, 9). However, it was not known whether the pineal gland produces neurosteroids until recently. We have recently demonstrated that the pineal gland is a newly found neurosteroidogenic organ producing a variety of neurosteroids from cholesterol (6, 7) (Figure 1).

Pregnenolone is an anabolic intermediate of most endogenous steroid hormones and is produced from cholesterol through the mitochondrial cholesterol side chain cleavage enzyme cytochrome P450scc (P450scc; encoded by the *Cyp11a* gene). We have demonstrated by transcription-polymerase chain reaction (RT-PCR) that the pineal gland in juvenile birds expresses *P450scc* mRNA (6, 7) (Figure 1). The protein product of this mRNA is localized in the cells that form the follicular structures in the pineal glands of birds (7). We have demonstrated by high-performance liquid chromatography (HPLC) with radioactive flow detector analysis that ^3^H-cholesterol is converted to radioactive pregnenolone when incubated with pineal gland extract from juvenile birds (6, 7). This observation has confirmed the presence of functional P450scc in the pineal gland (Figure 1), which has also been detected by gas chromatography-mass spectrometry (GC/MS) (7). Subsequent RT-PCR–based assessment has revealed that key steroidogenic enzymes, cytochrome P450 7α-hydroxylase (P4507α; encoded by the *Cyp7b* gene), 3α-hydroxysteroid dehydrogenase/Δ^5^-Δ^4^-isomerase (3α-HSD; encoded by the *Hsd3a* gene), 3β-hydroxysteroid dehydrogenase/Δ^5^-Δ^4^-isomerase (3β-HSD; encoded by the *Hsd3b* gene), 5α-reductase (encoded by the *Srd5a* gene), 5β-reductase (encoded by the *Srd5b* gene), cytochrome P450 17α-hydroxylase/c17,20-lyase (P45017α,lyase; encoded by the *Cyp17* gene), 17β-hydroxysteroid dehydrogenase (17β-HSD; encoded by the *Hsd17b* gene), and cytochrome P450 aromatase (P450arom; encoded by the *Cyp19* gene), are expressed in the pineal gland of birds (6, 7) (Figure 1).

We further demonstrated that steroid hormones are indeed present in the pineal gland. Incubation of ^3^H-pregnenolone with pineal glands from posthatch birds generates 7α- and/or 7β-hydroxypregnenolone by the action of P4507α found in the pineal glands (7) (Figure 1). In addition to these neurosteroid isomers, progesterone, allopregnanolone (3α, 5α-THP) and/or epipregnanolone (3β, 5β-THP), androstenedione, testosterone, 5α- and/or 5β-dihydrotestosterone, and estradiol-17β are also produced (7) (Figure 1). These *ex vivo* observations have confirmed that the pineal glands in juvenile birds have the biosynthetic machinery for major steroid hormones, which have also been verified to be produced as neurosteroids *in vivo* (7) (Figure 1). Although HPLC analysis has failed to resolve the isomers of these hormones, such as 7α-/β-hydroxypregnenolone, allo/epipregnanolone, and 5α-/β-dihydrotestosterone, several sets of isomers have been successfully isolated by GC/MS analysis (7). Especially, 7α-hydroxypregnenolone and allopregnanolone are actively released (6, 7).

Taken together, these findings indicate that the pineal gland in juvenile birds produces various neurosteroids from cholesterol. Accordingly, this is the first demonstration of neurosteroid synthesis in the pineal gland in a vertebrate.

## Physiological Function of Pineal 7α-Hydroxypregnenolone in Light-Dependent Locomotion

The chick pineal gland is used as a model for studies on the light-dependent phase-shifting mechanism of the circadian clock (10). To search for genes involved in this mechanism, a differential GeneChip analysis has been performed. This transcriptomics analysis has identified the light-induced transcriptional activation of the full set of genes in the pineal gland involved in cholesterol biosynthesis (6). When the pineal gland was exposed to light, it produced cholesterol and 7α-hydroxypregnenolone *ex vivo*. Interestingly, this light-induced production of 7α-hydroxypregnenolone occurred only when the gland was exposed to light at early night but not at late night or during the daytime. During early night time, the circadian clock is sensitive to light, which causes phase-delay of the clock (10). Thus, the light-sensitive pineal production of 7α-hydroxypregnenolone appears to be regulated by the circadian clock.

In vertebrates, an intracerebroventricular injection of 7α-hydroxypregnenolone activates locomotor activities (11–15). Thus, the intracerebroventricular injection of 7α-hydroxypregnenolone was administered in a dose-dependent manner at early night in chicks (6). After the injection, chicks were placed individually for locomotor activity measurement in an open field apparatus for 20 min. Spontaneous locomotor activities of chicks were stimulated by the intracerebroventricular injection of 7α-hydroxypregnenolone in a dose-dependent manner (6). Furthermore, when chicks are exposed to light during early night time, their locomotor activities reach the daytime level (6). These results suggest that pineal 7α-hydroxypregnenolone reaches the target sites within the brain by volume transmission (16) upon light exposure at early night.

## Physiological Function of Pineal Allopregnanolone in Purkinje Cell Survival During Development

7α-Hydroxypregnenolone and allopregnanolone are actively released during early posthatch period compared with adulthood (7). Therefore, 7α-hydroxypregnenolone and allopregnanolone may play key roles in birds during early posthatch period. In vertebrates, pinealectomy decreases cell number in the developing brain (17, 18). These findings suggest that these major neurosteroids secreted from the pineal gland are involved in the development of brain cells.

In chicks, pinealectomy decreases the concentration of allopregnanolone and the number of cerebellar Purkinje cells, whereas the supplementation of allopregnanolone to pinealectomized birds increases the concentration of allopregnanolone and recovers the number of Purkinje cells (7). Thus, pineal allopregnanolone is considered to be an essential factor for the normal development of cerebellar Purkinje cells. It thus appears that pineal allopregnanolone functions as an essential factor for Purkinje cells during posthatch period.

**Figure 2:**
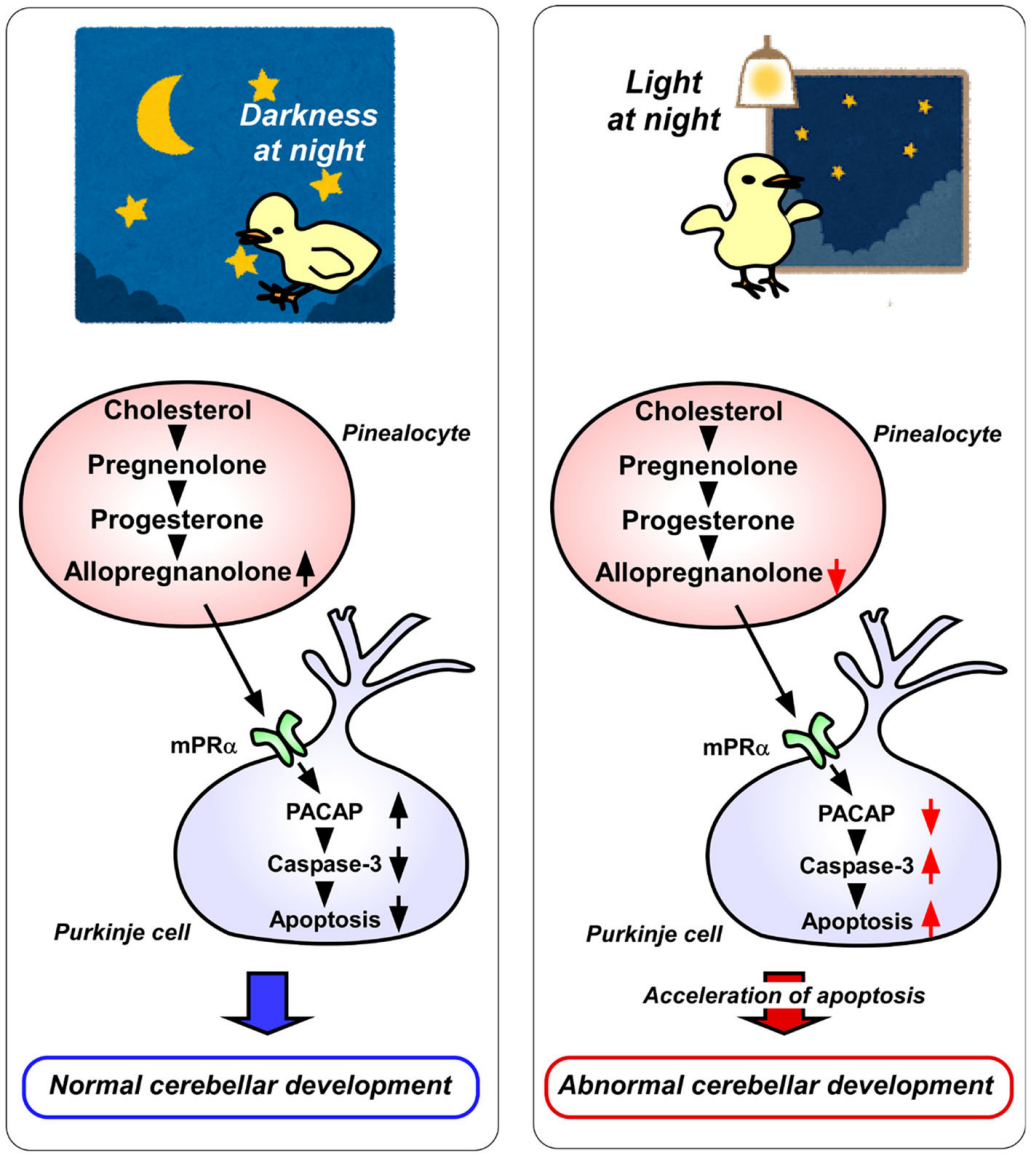
A schematic model of the effect of pineal allopregnanolone on Purkinje cell survival immediately after hatching under a 12/12 h light/dark cycle or with 1 h light exposure during the dark period (light-at-night condition). **(Left)** panel The normal cerebellar development under a 12/12 h light/dark cycle during the first week after hatching. Pineal allopregnanolone induces the expression of pituitary adenylate cyclase-activating polypeptide (PACAP), a neuroprotective factor, through the membrane progestin receptor α (mPRα) receptor binding mechanism in Purkinje cells. Subsequently, PACAP inhibits the activation of caspase-3 that facilitates the apoptosis of cerebellar Purkinje cells. **(Right)** panel The abnormal cerebellar development under the light-at-night condition during the first week after hatching. The light-at-night condition disrupts the diurnal rhythm in pineal allopregnanolone synthesis. Decreased pineal allopregnanolone synthesis leads to decreased expression of PACAP in Purkinje cells. Consequently, the active caspase-3 level increases, inducing the apoptosis of Purkinje cells in the cerebellum.

In addition, pinealectomy in juvenile birds increases the expression of active caspase-3 in Purkinje cells, whereas allopregnanolone supplementation decreases the expression of active caspase-3 during posthatch period (7). Thus, the neuroprotective action of pineal allopregnanolone on cerebellar Purkinje cells is exerted by suppressing the activation of caspase-3 (Figure 2).

Allopregnanolone acts mainly as a ligand of the γ-aminobutyric acid type A (GABA_A_) receptor and may also act as an agonist of the membrane progesterone receptors α (mPRα), as well as the mPRβ and mPRγ (19–21). Therefore, either mPR siRNA or isoallopregnanolone, an antagonist of allopregnanolone, was delivered into the cerebellum of posthatched chicks. It was found that the silencing of mPRα increases the number of Purkinje cells that express active caspase-3 in the cerebellum of chicks (8). Furthermore, to uncover the mechanism of neuroprotective action of allopregnanolone in cerebellar Purkinje cells, allopregnanolone action on the expression of neuroprotective/neurotoxic factors (22–26) has been investigated. Pinealectomy decreases the mRNA levels of pituitary adenylate cyclase-activating polypeptide (PACAP), a neuroprotective factor, in the cerebellum of juvenile birds (8). It has been found that a daily injection of allopregnanolone in pinealectomized juvenile birds upregulates PACAP relative to the levels in control birds (8). These findings show that PACAP mediates the neuroprotective action of pineal allopregnanolone through mPRα receptor binding during cerebellar development (Figure 2).

## Light-at-Night Affects the Development of Cerebellum Through a Mechanism Mediated by Pineal Allopregnanolone Action

It is known that environmental stimuli affect the development of animals including humans. In vertebrate brain development, a natural light-dark cycle promotes better brain development than constant conditions, such as constant light or constant darkness (27–31). However, the molecular mechanisms that control how environmental light conditions affect brain development remain unclear. The pineal gland is a photosensitive organ. To investigate whether light conditions are involved in the synthesis of allopregnanolone in the pineal gland, the birds have been incubated under either a 12/12 h light/dark (LD) cycle or LD cycle with 1 h light exposure during the dark period (light-at-night). Consequently, it has been found that the allopregnanolone concentration and synthesis during the dark period are higher in the pineal glands of LD birds than in those of light-at-night birds (8) (Figure 2). Furthermore, the number of cerebellar Purkinje cells is decreased by the light-at-night condition (8) (Figure 2). It is therefore considered that pineal allopregnanolone is a critical metabolite that affects cerebellar development in vertebrates, depending on the environmental light conditions.

## Conclusions

This review summarized the recent data on pineal neurosteroids. Studies have indicated that the pineal gland produces neurosteroids from cholesterol in birds. Pineal 7α-hydroxypregnenolone regulates locomotion in response to light stimuli in birds. Pineal allopregnanolone prevents the death of developing Purkinje cells by suppressing neuronal apoptosis during development. In addition, circadian disruption by light exposure during nighttime leads to cell death of developing Purkinje cells through pineal allopregnanolone-dependent mechanisms in juvenile birds. These observations suggest that nighttime artificial light exposure in modern societies may also perturb the development of the human brain.

Almost all animals have circadian rhythms. However, modern life conditions chronically disrupt circadian rhythm through artificial light exposure. The disruption of circadian rhythm is associated with a decline in mental and physical health (32–34). The most potent circadian rhythm disruption is inappropriately timed bright light exposure (e.g., light-at-night). To investigate the effects of chronic circadian disruption in modern societies on mental and physical health, which is efficiently modeled by the light-at-night condition presented here, many studies have been conducted on mice. However, it is important for us to bear in mind that laboratory mice are mainly nocturnal animals, whereas humans are diurnal. Thus, birds are excellent animal models to uncover the effect of light-at-night on diurnal animals, including humans.

## Author Contributions

SH and KT wrote the manuscript. All authors contributed to the article and approved the submitted version.

## Conflict of Interest

The authors declare that the research was conducted in the absence of any commercial or financial relationships that could be construed as a potential conflict of interest.
